# Treatment Approaches to Moderate to Severe Psoriasis

**DOI:** 10.3390/ijms18112427

**Published:** 2017-11-16

**Authors:** Paolo Gisondi, Micol Del Giglio, Giampiero Girolomoni

**Affiliations:** Department of Medicine, Section of Dermatology and Venereology, University of Verona, 37129 Verona, Italy; micol.delgiglio@univr.it (M.D.G.); giampiero.girolomoni@univr.it (G.G.)

**Keywords:** psoriasis, therapy, biologics

## Abstract

Psoriasis is a common disease, which has a considerable impact on patients and the health care system. Treatment approaches to the disease may be various because some issues are not definitely addressed. Moreover, the therapeutic paradigms are continuously changing because of the recent approval of new treatments for psoriasis such as interleukin (IL)-17 inhibitors and apremilast. In this review, the factors influencing psoriasis severity, the indications for systemic treatments, the overall parameters to be considered in the treatment choice, life style interventions, and the recommendations for the use, screening, and monitoring of systemic therapies available including acitretin, cyclosporine, methotrexate, apremilast, adalimumab, etanercept, infliximab, secukinumab, ixekizumab, and ustekinumab are discussed. Finally, treatment approaches in special patient populations including children, the elderly, pregnant women, patients with a history of neoplasm, and candidates for surgical procedures are reported.

## 1. Factors Influencing Psoriasis Severity

Factors influencing psoriasis severity include the extent of disease, the location of lesions, the degree of inflammation, the responsiveness to treatment, and the impact on quality of life. Most of the definitions of disease severity have been developed as inclusion criteria of patients in randomized controlled clinical trials. The severity of chronic plaque psoriasis is generally assessed according to the Psoriasis Area and Severity Index (PASI), the body surface area (BSA), and the Physician Global Assessment (PGA) [1]. A patient’s quality of life is commonly assessed by questionnaires including the Dermatology Life Quality Index (DLQI) and the Short Form (SF-36) Health Survey [2]. According to the European S3 Guidelines on the systemic treatment of psoriasis vulgaris, moderate to severe disease is defined as a PASI score >10 [3]. In line with this definition, the “Rule of Tens” defines a patient’s disease as severe if any one of the following criteria is met including PASI ≥10 or DLQI ≥10 or BSA ≥10% [4]. However, the involvement of visible areas such as the face, scalps, hands, and nails or the presence of severe symptoms including itching that is not properly topically treated may require systemic treatment [5]. Consequently, in clinical practice, a systemic therapy could be indicated even if PASI or BSA is lower than 10.

## 2. Treatments Goals 

**Figure 1:**
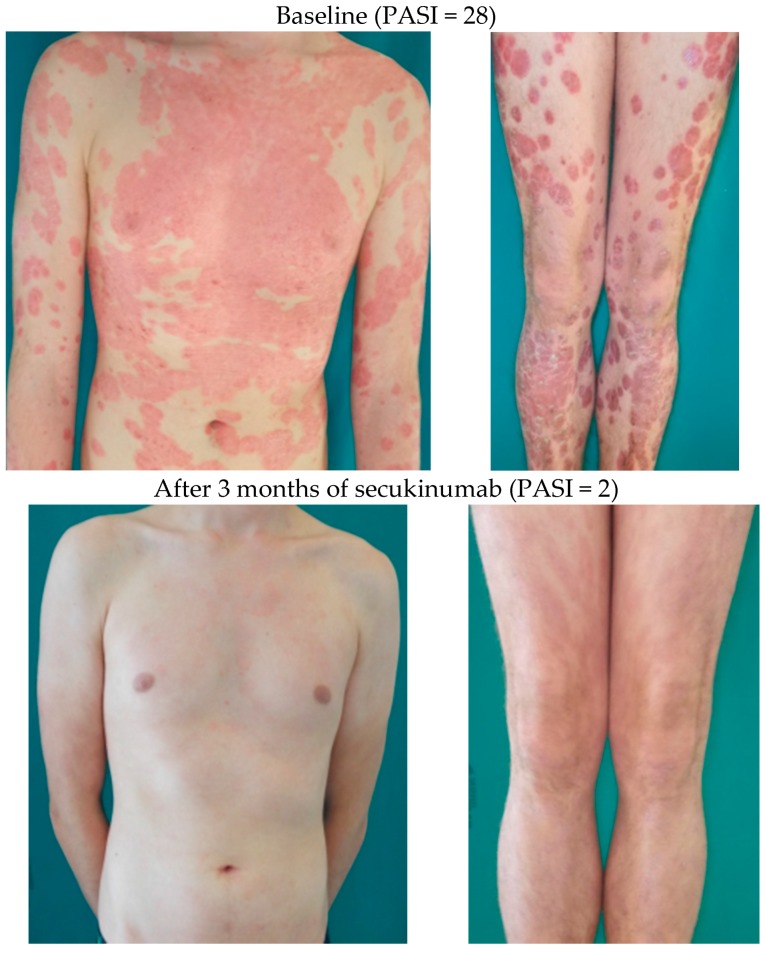
An 18-year-old man with severe chronic plaque psoriasis. Otherwise healthy, no comorbidities and concomitant medications. Treated in the past with topicals, as well as phototherapy (nb-UVB), methotrexate, acitretin, and cyclosporine with no or very limited efficacy. He had manifested good but unsatisfactory response to adalimumab (PASI50). Treatment with secukinumab led to a dramatic response within two months (PASI90).

Treatment goals have been agreed to decide when and how to progress along treatment algorithms [5]. Treatment goals are based on a selected list of outcome measures that consider not only the severity of skin symptoms but also the impact of disease on the quality of life. In Europe, the percentage reduction in the PASI score is the standard measure of assessing the decrease in psoriasis severity and consequently the effectiveness of treatments. The PASI75 response is the proportions of those who have reached 75% amelioration in their baseline score. PASI75 is the current benchmark of primary endpoints for most clinical trials of psoriasis [6]. However, the ultimate goal of therapy is the complete or almost complete clearing of skin lesions, and an improvement of 90% or greater (PASI90 response) is currently considered as the most relevant treatment outcome, especially in patients with severe disease [7,8]. Indeed, investigator global assessment equal 0 or, i.e., “clear” or “almost clear”, is commonly used as co-primary end point together with PASI90 in clinical trials. If the patient does not improve by at least 50% in the PASI score, s/he is considered a non-responder [5]. Treatment goals are generally evaluated between Weeks 12 and 16 at the end of the induction therapy. If the goal is not achieved, several strategies can be adopted such as raising the dose of the drug, reducing the time gap between administrations, or combining the drug with another drug. Changing the drug is indicated when the mentioned adjustments are ineffective or inappropriate (Figure 1) [9]. 

## 3. Indications for Systemic Therapy 

Treatments of psoriasis can be classified as topical, systemic, or phototherapeutic. Topical therapy alone is indicated in mild psoriasis. The adherence to topical therapy in patients with psoriasis is generally low, particularly in the long term, and this could impair its effectiveness in real life [10]. For patients with moderate to severe psoriasis, the topical therapy could be indicated in association with systemic treatments. The indications for systemic treatment are the following: a PASI greater than 10; a PASI less than 10 but with the involvement of scalp, face, hands, nails, palmoplantar, or genital area; psoriasis associated with severe symptoms that are not controlled by topical treatment; severe impact of the disease on quality of life (e.g., DLQI ≥10). Lastly, the presence of an active psoriatic arthritis could require a systemic treatment independently by the PASI score. 

Several parameters should be considered in the choice of treatment including the characteristics of the disease (e.g., severity and location of skin lesions), patient-related features (e.g., age, previous treatment failures), and the characteristics of the treatments (e.g., efficacy and safety issues). Some considerations can be addressed in the choice of the biologic drug. A history of latent tuberculosis, severe heart failure, demyelinating disease, or alopecia areata may contraindicate or raise caution on the use of tumor necrosis factor (TNF)-α blockers. The concomitance of an inflammatory bowel disease such as Crohn’s disease is a relative contraindication for IL-17 inhibitors, whereas it may favor the choice of anti TNF-α monoclonal antibodies that are indicated in both disorders.

## 4. The Long-Term Management of Psoriasis 

Currently we have only limited data on the natural history of psoriasis and on factors predicting its prognosis. Psoriasis is a life-long disease with a chronic relapsing course, and most patients require a long-term management. The disease’s evolution is unpredictable and the extent of skin involvement can range from mild to very severe forms. The interval of time between the episodes of psoriasis recurrence may vary from a few months to several years. Remission of psoriasis is achieved in the long-term efficacious control of skin lesions. The long-term use of conventional systemic treatments is limited mostly by poor tolerability and cumulative toxicity including liver toxicity from methotrexate, renal toxicity from cyclosporine, and skin carcinogenesis from phototherapy, particularly psoralens plus ultraviolet A (PUVA). Phototherapy including narrow band ultraviolet B (UVB) and PUVA has many biological effects that exert an anti-psoriatic action including reduced mobility of antigen-presenting Langerhans’ cells, inhibition of T-cell activation, induction of apoptosis in activated T cells, anti-proliferative effects on keratinocytes, and anti-angiogenic effect and endothelial cells [3].

In contrast, long-term use of biologics is more feasible, because of better tolerability and safety. An increased risk of overall infections including herpes zoster and Candida infections is the only major concern associated with TNF-α and IL-17 inhibitors, respectively [11,12]. Generally, the continuous regimen of biologics has greater efficacy and safety compared to an intermittent regimen [13]. Indeed, the risk of adverse effects and/or developing antidrug antibodies is significantly higher in those patients receiving biologics as an intermittent regimen. It has not been fully clarified whether stopping biological therapy is appropriate in those patients who achieve continuing psoriasis remission. An international consensus agreed that stopping biologic therapy is not generally recommended [5]. However, if agreed to by the patient (and after achieving complete remission for a minimum of 1 year), stopping biologic therapy can be considered with careful follow-up. Subgroups in which stopping therapy might be considered are those patients who clearly require treatment interruption, in the absence of comorbidities including psoriatic arthritis (PsA), and in the case of low impairment of quality of life. Lastly, there are a few biomarkers capable of predicting treatment outcome. High body mass index predicts poor response and long-term efficacy to conventional and biological treatments [14]. Specific TNFAIP3 single nucleotide polymorphisms have been associated with a higher response rate to etanercept and adalimumab [15]. Similarly, IL-17A and IL-17F single nucleotide polymorphisms predict a higher response to ustekinumab, infliximab, or adalimumab [16]. A faster and higher response to ustekinumab has been confirmed in HLA-Cw6-positive patients [17]. 

## 5. Non-Pharmacological Treatment Approaches

The association between psoriasis severity and metabolic comorbidities, anxiety, depression, smoking, and alcohol abuse has been confirmed in several studies. Despite these strong associations, limited data are available on the impact of non-pharmacological interventions on psoriasis. It has been recommended that blood pressure, body mass index, waist circumference, smoking, alcohol consumption serum lipids, and fasting glucose should be regularly assessed in patients with psoriasis, particularly in those with the more severe form [18]. Moreover, metabolic disorders and cardiovascular morbidity has also been associated with vitamin D insufficiency, which may be also associated with psoriasis [19]. Moreover, low levels of vitamin D may also have important implications in the pathogenesis of psoriasis. Vitamin D regulate keratinocyte growth and differentiation, as well as immune functions of dendritic cells and T lymphocytes. Topical vitamin D derivatives are extensively used as monotherapy or in combination with steroids for the topical treatment of psoriasis.

### 5.1. Smoking Cessation Interventions

Cigarette smoking is recognized as the single most important source of preventable mortality, particularly for cardiovascular disorders. However, there is no robust evidence to support the impact of quitting smoking on psoriasis outcome [20]. 

### 5.2. Weight Reduction Interventions

Evidence is growing that body weight reduction could decrease the severity of psoriasis and increase the response to systemic treatments in overweight or obese patients [21]. Moreover, obesity can affect both drug’s pharmacokinetics and pharmacodynamics, leading to a reduction in the treatment response. Referral to a dietician or bariatric surgery could be indicated for those obese patients who are motivated to lose weight. Moreover, the healthy eating pattern, such as the Mediterranean diet, should be favored because nutrition may play a major role in psoriasis. Low adherence to the Mediterranean diet has been associated with psoriasis severity [22]. In addition, alcohol use disorders including heavy alcohol consumption is more prevalent in patients with psoriasis, and it has been associated with disease severity. Specific interventions to reduce alcohol abuse in these patients are appropriate [23].

### 5.3. Interventions on Psychiatric Comorbidities

Limited evidence from small randomized trials suggests the effectiveness of interdisciplinary care involving psychiatric support on the quality of life of psoriatic patients [24]. Given the bearing of psychiatric comorbidities on quality of life and their impact on disease management, patients with psychological distress or symptoms of depression should be offered referrals to a psychiatrist and psychological support. 

## 6. Treatment Approaches in Special Patient Populations

### 6.1. Systemic Therapy of Psoriasis in Children

Psoriasis during infancy, childhood, or adolescence could have a significant impact on patient and family quality of life, and those young patients who cannot be managed effectively with topical treatment should be considered for systemic therapy. Most current systemic treatments for psoriasis are off-label in children, such as acitretin, cyclosporine, methotrexate, infliximab, ixekizumab, secukinumab, and apremilast. However, methotrexate, cyclosporine, and acitretin have been used in pediatric patients, and their effectiveness and safety data are available and derived mainly from by case series studies. In contrast, etanercept, adalimumab, and ustekinumab have been approved for psoriasis vulgaris in children and adolescents because randomized controlled clinical trials have been performed. 

### 6.2. Systemic Therapy of Psoriasis in the Elderly 

Systemic therapy of psoriasis in the elderly is becoming important issue because of the increase in the aged population. When selecting a treatment for an elderly patient, dermatologists should consider the possible concomitance of comorbidities including cardiometabolic disorders and chronic kidney disease, because they could contraindicate the prescription use of some conventional treatments. Moreover, the use of concomitant medications for comorbidities could be at risk for potentially harmful drug interactions. In addition, there is a scarcity of data in the literature regarding the treatment of psoriasis in the elderly population because they are generally excluded form randomized clinical trials [25]. 

### 6.3. Pregnancy 

The treatment of moderate to severe psoriasis in pregnancy is challenging. Mid-potency topical corticosteroids for limited periods are the preferred topical therapy for mild disease. Long-term use of potent or very potent steroids during pregnancy may be associated with low birth weight [26]. Tazarotene is contraindicated in pregnancy because it is a teratogen. Salicylic acid, coal tar, and calcipotriol are best avoided. The calcineurin inhibitor tacrolimus may be used in sensitive areas such as face and skin folds where no other alternative exists, since systemic adsorption is very low. nb-UVB phototherapy is considered safe and useful to treat pregnant patients, whereas PUVA therapy is contraindicated because psoralens are a known mutagen [27]. Retinoids are teratogen, so they are pregnancy category X, i.e., contraindicated during pregnancy. Fertile female patients need to avoid conception for a minimum of two years after acitretin withdrawal. Methotrexate is also a pregnancy category X [28]. Female but also male patients need to avoid conception for a minimum of three months after methotrexate withdrawal. Cyclosporine is in pregnancy category B, and has been proved not to be a teratogen with successful pregnancy outcomes in transplanted patients and those affected by inflammatory bowel diseases [29]. Similarly, biologics are in pregnancy category B [30]. However, biologics may be used only in high-need situations and when no alternative treatments are available. Alternative treatments should be considered throughout pregnancy, with nb-UVB phototherapy as the preferred therapy. 

### 6.4. Patients with a History of Neoplasm

Patients with a positive history of neoplasm demand a cautious prescription of systemic drugs that could potentially increase the risk of recurrence. Topical therapy, phototherapy acitretin, and/or methotrexate are advisable for patients with recent malignancy (i.e., within 5 years) as a first line treatment approach. Cyclosporine is not recommended, and phototherapy is not indicated in patients with cutaneous malignancy including melanoma. In the case of inadequate response to first line treatment mentioned, the decision to initiate biologics or apremilast should be discussed with oncologist. Type and staging of cancer, the risk of recurrence and the burden of psoriasis represent the most important elements to be considered [31]. 

### 6.5. Patients Undergoing Surgical Procedures

It has been reported that continuing biologic treatments in psoriatic patients undergoing surgical procedures did not increase the risk of post-operative complications, while perioperative withdrawal of biologic therapy could increase the risk of psoriasis flare [32,33]. In the case of patients that are candidates for minor surgery such as dental procedures and skin surgery, any systemic treatment could be continued without any issue. Before major surgery (when the pleural cavity, the peritoneum, or meninges are opened), systemic immunosuppressive treatment should be withdrawn and restarted if postoperative infection is excluded. 

## 7. Conclusions

Psoriasis is a common and chronic skin disorder. Its pathogenesis derives from a combination of genetic and environmental risk factors. Psoriasis is T cell-driven, with the Th1 and Th17 cells playing a pivotal role by producing different cytokines including interleukin (IL)-6, IL-17, and IL-22, interferon (IFN)-γ, and tumor necrosis factor (TNF)-α, resulting in increased proliferation of epidermal cells, neo-angiogenesis, and infiltration of white cells in the skin. Moreover, it has been reported that de novo glucocorticoids synthesis is dysfunctional in non-lesional and lesional psoriatic keratinocytes, and this could represent an additional pathological mechanism in the psoriasis pathogenesis [34] The clinical presentation of chronic plaque psoriasis can widely vary among patients because of different ages of onset, types of symptoms, areas of involvement, and disease severity. Approximately one-third of patients with psoriasis is affected by PsA. In addition, obesity, diabetes, fatty liver disease, metabolic syndrome, and cardiovascular disorders are frequently associated with moderate to severe psoriasis. In addition, there is a special population of patients, such as children, the elderly, pregnant women, patients with a history of neoplasm, and those who are candidates for surgical procedures, who need specific consideration in the cautious use of treatments. Consequently, the treatment approach should be personalized according to the specific characteristics and needs of the patient. Psoriasis is a life-long disease that requires long-term treatment. The long-term use of conventional systemic treatments is limited mostly by poor tolerability and cumulative toxicity. In contrast, the long-term use of biologics is more advisable, because of better tolerability and safety, although their cost is an issue for their sustainability. The issue of long-term sustainability may be partially managed with the use of biosimilars because they are cheaper. A biosimilar is a biological drug, which is similar to a reference product that is already approved and is expected to have the same safety and efficacy profile. At this stage, only biosimilars of infliximab and etanercept are available, but the adalimumab biosimilar will be soon. Finally, non-pharmacological lifestyle interventions such as body weight reduction in obese patients are being considered relevant in the global management of patients.

## Abbreviations

PsA	psoriatic arthritis
UVB	ultraviolet B
nb	narrow band
PUVA	psoralen and ultraviolet A
IL	interleukin
IFN	interferon
TNF-α	tumor necrosis factor-α
